# Waste Tyres Pyrolysis for Obtaining Limonene

**DOI:** 10.3390/ma13061359

**Published:** 2020-03-17

**Authors:** Katarzyna Januszewicz, Paweł Kazimierski, Wojciech Kosakowski, Witold M. Lewandowski

**Affiliations:** 1 Department of Energy Conversion and Storage, Faculty of Chemistry, Gdańsk University of Technology, G. Narutowicza 11/12, PL-80-233 Gdańsk, Poland; wlew@pg.edu.pl; 2 Institute of Fluid-Flow Machinery, Polish Academy of Sciences, Fiszera 14 st., PL-80-231 Gdańsk, Poland; pawel.kazimierski@imp.gda.pl; 3 Polmos Żyrardów Sp. z o.o. (ul. Mickiewicza 1-3), PL-96-300 Żyrardów, Poland; wkosakowski@poczta.onet.pl

**Keywords:** pyrolysis tyres, waste tyres, limonene obtaining

## Abstract

This review deals with the technologies of limonene production from waste tyre pyrolysis. Thermal decomposition is attractive for tackling the waste tyre disposal problem, as it enables both: energy to be recovered and limonene to be obtained. This material management recycling of tyres is environmentally more beneficial than the burning of all valuable products, including limonene. Given this recoverability of materials from waste tyres, a comprehensive evaluation was carried out to show the main effect of process conditions (heating rate, temperature, pressure, carrier gas flow rate, and type of volatile residence and process times) for different pyrolytic methods and types of apparatus on the yield of limonene. All the results cited are given in the context of the pyrolysis method and the type of reactor, as well as the experimental conditions in order to avoid contradictions between different researchers. It is shown that secondary and side reactions are very sensitive to interaction with the above-mentioned variables. The yields of all pyrolytic products are also given, as background for limonene, the main product reported in this study.

## 1. Introduction

We have reviewed all the publications related to the pyrolysis of used tyres and rubber waste that we were able to access in order to extract information on reactor design, operating principles, technological procedures and mass yields of pyrolytic products, i.e., oil, gas, residual solids and their proportions. The comparison of those results and type of reactors were compered elsewhere [1]. However, even in these publications, due to limited scope, we were unable to include considerations of limonene. The issues relating to limonene not treated in [1] are therefore examined in the present paper.

Limonene and other monoterpenes are widely used in the production of resins, gums, lacquers, printing inks and perfumes. Notably, 30,000 tons of ***α***- and ***β***-pinenes are used every year in the perfumery, cosmetics, food and pharmaceutical industries alone. The demand for monoterpenes is therefore great, so for researchers and technologists they are also an important object of study.

Although limonene is used directly as a natural aroma in essential oils or perfumes, its main value is as a precursor for the chemical synthesis of its oxidized derivatives, usually compounds with the same carbon skeleton as limonene. The main oxygenated derivatives of limonene are menthol, carvone, carveol, ***α***-terpineol, limonene oxide, perillyl alcohol; because of their medicinal properties, they are generally more expensive than limonene itself. Limonene, and its derivative perillyl alcohol in particular, are used preventively and therapeutically in oncology, as they inhibit the growth of liver, skin, breast, lung, pancreas, colon and prostate cancers [2,3,4,5,6]. The nature of this effect is based on three mechanisms: a) modification of p21 *ras Oncoproteins*, which are responsible for the intracellular transmission of signals of tumor cell division; b) inhibition of the synthesis of coenzyme Q, in the absence of which tumor cells are easier to break down oxidatively; and c) activating the expression of transforming growth factor-***β*** [7,8,9,10,11].

In addition, compounds such as limonene and pinene may be excellent starting materials for the industrial synthesis of many other valuable compounds, the production costs of which are much lower than when they are manufactured from basic materials, such as the distillation products of crude oil [12,13]. Menthol, a derivative of limonene, which is known for its flavor qualities and being an excellent anesthetic, used to alleviate irritation of the mucous membranes and in the supportive treatment of colds, is more than 30 times more expensive than limonene [14]. The properties of ***d***-carvone resemble those of menthol: it, too, stimulates the nervous system, and in addition, can inhibit the sprouting of stored potatoes.

The hydrophobic properties of monoterpenes enables them to be used in, for example, the non-invasive, natural dissolution of gallstones [7]. Para-cymene, formed by the dehydrogenation of limonene, is also a very important intermediate, used in the production of p-cresol. All of these compounds, together with limonene and its oxidized forms, are components of essential oils and are used as flavors or aromas in the food, perfume and cosmetics industries [12,15]. In addition, the highly reactive limonene oxide is a raw material for the synthesis of the more complex compounds necessary for the production of drugs, plastics, etc. [12,16].

## 2. Properties and Characteristics of Limonene

### 2.1. Chemical Structure of Limonene 

**Figure 1:**
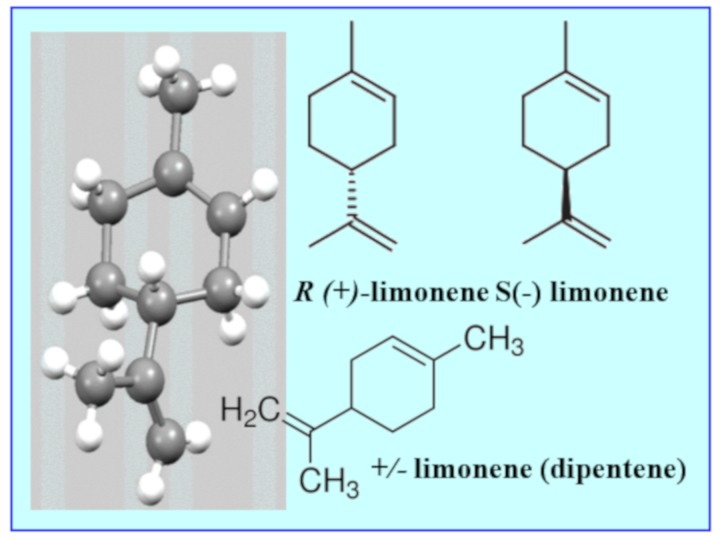
Structural formulas of limonene and its enantiomers.

Limonene is classified as a cyclic monoterpene [17] with the chemical formula C_10_H_16_ and the IUPAC name 1-methyl-4-(1-methylethenyl)-cyclohexene. Its common name derives from lemons: limonene is present in the skins of these fruit and is responsible for their distinctive smell. It is derived from isoprene and is present, together with other terpenes, in the oil obtained from the pyrolysis of polyisoprene, or natural rubber, the main component of truck tyres. Limonene is a chiral compound, i.e., it has an asymmetric center in which the carbon atom is linked to four different substituents (Figure 1). This property determines the formation of its two optical isomers: dextrorotatory ***d*** or (+) limonene (citrus, cumin, celery) and levorotatory ***l*** or (-) limonene (mint oil, conifer trees). Limonene also occurs as a racemic mixture known as dipentene (camphor, bergamot) [15].

The more common isomer—***d***-limonene—smells strongly of citrus fruits and has antioxidant, antibacterial and anti-cancer properties. Used in chemical synthesis as a precursor of carvone and as a renewables-based solvent in cleaning products, it has an annual production of about 73,000 tons, mainly by extraction from the skins of citrus fruits.

The less common ***l***-isomer of limonene is found in mint oils and has a piney, turpentine-like odor.

Bicas et al. [18] studied the biotransformation of ***d***-limonene into ***d***-***α***-terpineol with a floral scent, used in soaps, cosmetics and fragrance formulations. The fungi and molds *Cladosporium* sp. [19], *Pseudomonas gladioli* [20] and *P. digitatum* [21] are used in its biotech production.

At higher concentrations, limonene, and especially its oxidized forms, are allergens, irritating the skin, mucous membranes and eyes. It is therefore subject to registration: it must be included on the INCI (International Nomenclature of Cosmetic Ingredients) list when the concentration of limonene >0.01% in rinseable products and >0.001% in products that remain on the skin.

Limonene, as mentioned in the introduction, is widely used in the perfumery, cosmetics, food and pharmaceutical industries alone. In addition, it is a precursor for the synthesis of many other organic compounds, such as menthol, carvone, carveol, α-terpineol, limonene oxide, perillyl alcohol, which, due to their aroma and medicinal properties, are generally more expensive than limonene itself. Technical limonene (a mixture of d and 1 limonene), together with other terpenes, is also an excellent solvent used in the plastics, paints, and varnishes, automotive and construction industries.

### 2.2. Physical Properties of Limonene

Limonene is a colorless, flammable and toxic liquid with a characteristic smell of lemons. It has the following physical properties: melting point: −74.35 °C, boiling point: 176 °C, vapor pressure: 300 hPa at 50 °C and 2.1 hPa at 20 °C, flash point: 43 °C (DIN 51758), auto-ignition temperature: 225 °C, std. enthalpy of combustion:−6.128 MJ/mol, upper and lower explosion limit in air: 0.7 vol.% and 6.1 vol.%, density: 841.1 kg/m^3^; insoluble in water, but miscible in alcohol, benzene, chloroform, ether, CS_2_ and oils soluble in CCl_4_ [22].

## 3. Thermal Breakdown Mechanism of Natural Rubber

Pyrolysis is the thermal anaerobic decomposition of organic compounds into less complicated ones with lower molecular weights. The products of gaseous pyrolytic decomposition include mainly short-chain hydrocarbons, along with CO, CO_2_ and H_2_, longer-chain aliphatic and aromatic hydrocarbons, which make up the main component of the oil fraction, and a solid residue—mainly char and ash.

**Figure 2:**
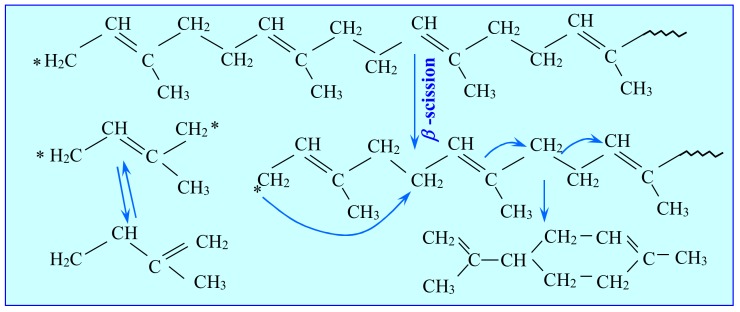
Mechanism of the pyrolytic decomposition of natural rubber to limonene and isoprene monomers [23].

**Figure 3:**
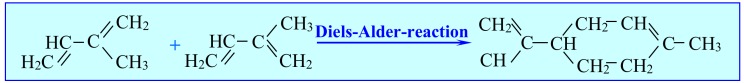
The second pathway of limonene formation from isoprene monomers via the Diels- Alder reaction.

Natural rubber is a polymer consisting of chains of isoprene molecules. By the scission of these chains of polyisoprene and the tendency of the polymer radicals to form hexahydric ring compounds, limonene is one of its thermal decomposition products (Figure 2). In addition to the primary product—the limonene dimer—the pyrolysis of isoprene also generates monomers of isopentene, which via the Diels-Alder cyclization reaction, are a secondary source of ***dl***-limonene (Figure 3) [23] or [24].

**Figure 4:**
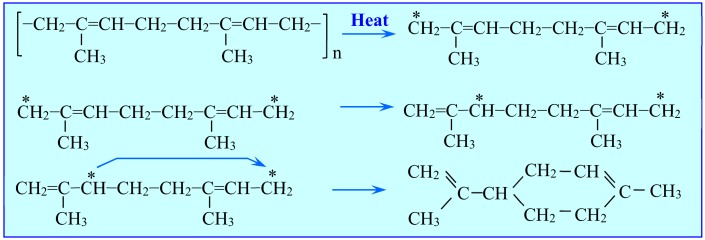
Mechanism of the pyrolytic decomposition of natural rubber (polyisoprene) to propylene and its isomerization into limonene [25].

A very similar mechanism of limonene production, namely the depolymerization of polyisoprene, cyclization of the resulting dimer radicals, and their two-step pyrolytic isomerization, is shown in Figure 4 [25]. The significant increase in the number of road vehicles worldwide means that the disposal of waste tyres has become a serious pollution problem. Since the calorific value of rubber and soot from tyres is higher than that of coal, it would seem reasonable to make use of their high energy potential by combusting them in industry, e.g., in cement works, brickworks, glassworks or porcelain factories. But the burning of tyres is environmentally harmful inter alia because of the sulfur they contain: the sulfur oxides (SO_x_) and other harmful contaminants like NO_x_ or VOCs emitted in the exhaust gases pollute the air. Likewise, the combustion of high-molecular-chain organic compounds that could otherwise be recycled into monomers and re-polymerized into new products is neither environmentally friendly nor economical. However, the ab initio synthesis of monomers for the manufacture of such products is less cost effective and more energy intensive than any gain made from the combustion of these polymers contained in tyres. The latter argument also applies to limonene, whose pyrolytics production from used tyres can be competitive and environmentally friendlier than other methods.

## 4. Classification of Tyre Pyrolysis Technology

As stated above, pyrolysis is a process in which organic substances are decomposed to lower-molecular weight products, liquids or tars, chars [26] and small amounts of gases (volatiles). Materials are heated in a reducing or non-oxygen environment, usually at a low (<400 °C), medium (400–600 °C) or high temperature (>600 °C). Various attempts have therefore been made to reduce the energy consumption of tyre pyrolysis [27,28,29,30,31]. The required thermal energy can be supplied in several ways, divided into two main categories: conventional (internal—partial combustion of the load, external—electrical resistance or flame [32,33]) and non-conventional (microwave [34,35,36,37,38,39], ultrasonic [40,41,42,43,44,45,46], plasma [47] and supercritical water [48,49,50,51] or carbon dioxide [52,53]).

To date, only four of the possible pyrolysis techniques with conventional (internal or external) heating for large-scale applications have been employed: gas–solid pyrolysis at temperatures of 450–500 °C ([27,54,55]), vacuum pyrolysis (<450 °C, [56,57,58,59,60]), liquid–solid pyrolysis (400–500 °C) [61] and catalytic pyrolysis (300–600 °C) [62,63,64,65,66,67,68]. Generally, pyrolysis leads to the total recovery of mass as solid (non-volatile material), liquid (condensable fraction) and gaseous (non-condensable fraction) products. The gas phase consists mainly of aliphatic hydrocarbons, hydrogen and hydrogen sulfide; the liquid phase contains primarily aromatic hydrocarbons; and the solid residue consists of steel, carbon black and unreacted organic matter (char) [69]. These three products are always obtained, regardless of apparatus, heating source, operating temperature and heating rate. The usual aim in all research is to enhance yields of the liquid products, rich of limonene, and to control product characteristics.

### 4.1. Vacuum Pyrolysis

The vacuum pyrolysis of waste tyres was first performed and described by Benallal et al. [70] At a maximum temperature *t* = 500 °C, an absolute pressure *p* = 13 kPa and a feed rate of 19 kg/h of cylindrical particles from cross ply tyres (*h* = 12 mm, *d* = 6 mm), they obtained ca 50% of hydrocarbon oil, 25% of carbon black, 9% of steel fibers and 5% of gases. The pyrolytic oil was distilled into four fractions: light (boiling range *t* < 160 °C; ca 20%), average (*t* = 160–204 °C; ca 6.8%), heavy (*t* = 204–350 °C ca 30.7%), and a residue (*t* > 350 °C; ca 42.5%).

**Figure 5:**
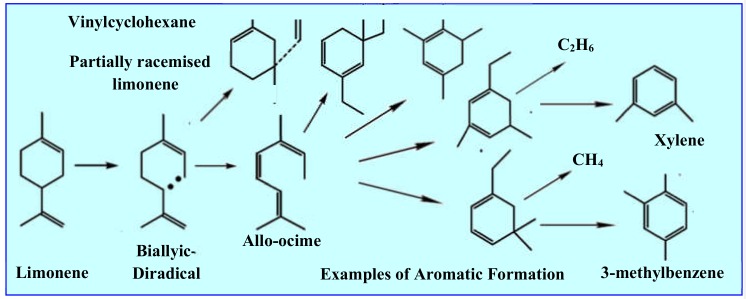
Mechanism of secondary limonene conversion (thermal degradation) into other aromatic derivatives of BTX (benzene, toluene, xylene). [23].

Vacuum pyrolysis, a novel approach to the low-temperature and reduced-pressure decomposition of organic compounds, has several advantages in comparison with atmospheric gas–solid pyrolysis. The most important is the shorter residence time of the liquid fraction in the reactor, which reduces the occurrence and intensity of secondary reactions in accordance with the mechanisms shown in Figure 5.

Another advantage is the higher yield of oil, and therefore of limonene. Temperature, pressure and organic vapor residence time in the pyrolizer are three important factors influencing the formation of pyrolysis products and their composition [70,71].

Further studies of vacuum pyrolysis of automobile tyres by Roy et al. [56,57,58,59,60,61,62,63,64,65,66,67,68,69,70,71,72,73,74,75,76,77,78,79,80,81] were conducted at a pressure of *p* < 10 kPa and over a temperature range of *t* = 480–520 °C. As a result, they obtained ca 47% of oil, ca 3% of gas, ca 39% of carbon black and ca 10% of steel wire residue.

Subsequent research into vacuum pyrolysis of tyres addressed the following aspects:

- the development of technologies for obtaining an active carbon black with reduced ash content and parameters similar to N-600 or N-700 carbon black, used as a filler in the tyre industry; char, the equivalent of activated carbon, which can be used as an adsorbent, catalyst, filler of PCV and other plastics, the raw material for the production of pigments for paints, varnishes and inks, and also a component of bitumen (5–15%);
- the application of pyrolysis oil, which can be added to gasoline (light fraction), used as a plasticizer in rubber mixtures (medium fraction) and as the raw material for the production of coke and asphalt (heavy fraction),
- finding a method of carrying out pyrolysis in order to increase the proportion of limonene in the oil, for example, by the addition of catalysts (Na_2_CO_3_, NaOH [23]). The possibility of obtaining limonene from tyre pyrolysis has proved to be so interesting that many publications have already been written on this topic, including the present article.

### 4.2. Gas–Solid Pyrolysis

In this system, pyrolysis involves the thermal decomposition of comminuted tyres in an atmosphere of inert non-oxidizing gas (nitrogen, helium, hydrogen, CO_2_, argon [82], exhaust or recycled pyrolysis gas). This gas prevents the pyrolizer content from burning and intensifies heat transfer. Gas–solid contact has been studied by many researchers, who have put forward different reactor technologies: fluidized bed, rotary kiln reactor, screw conveyor kiln reactor, fixed counter flow bed reactor, retort reactor [54,71,83,84,85,86,87].

The amount of oil obtained is inversely proportional to the temperature, residence time and inert gas pressure. When hydrogen is the inert gas, the oil is simultaneously hydrogenated. With other non-reactive gases, it was found that their kind and the pressure does not greatly affect the process, which takes place quickly and efficiently.

Paper [88] describes pyrolytic tyre decomposition in the temperature range *t* = 350–700 °C in the presence of N_2_, as a result of which ca 52% fraction of oil, ca 38% of char and ca 10% of gaseous products were obtained. The highest yield of oil was obtained at *t* = 560 °C, its main components being aromatic and aliphatic hydrocarbons and compounds containing hydroxyl groups. The gaseous fraction contained H_2_, CO, CO_2_ CH_4_, C_2_H_6_ and C_2_H_4_; the char, mainly coke, can be activated or used for the production of briquettes.

Papers [89,90] describe the pyrolysis of whole tyres in nitrogen at atmospheric pressure and at temperature *t* = 300–700 °C. The gaseous products contained light hydrocarbons (C_1_-C_4_), CO, CO_2_ and H_2_S, the liquid fraction contained a mixture of heavier hydrocarbons C_5_-C_20_, predominantly aromatics (approx. 75%), and the solid fraction contained carbon black, steel cord and ash. Distillation of the liquid fraction yielded light petroleum oil (20%, boiling point *t*_w_ < 160 °C), gasoline (10%, *t*_w_ = 160–204 °C) and the oil equivalent of diesel fuel (35%, *t*_w_ = 204–350 °C. The yield of aromatic (toluene, benzene, xylene, biphenyl, naphthalene) and polycyclic hydrocarbons in the liquid fraction initially increased with rising pyrolytic temperature, but at temperatures *t* > 500 °C, began to decrease.

The atmospheric pyrolysis of shredded tyres in helium was studied by Zabaniotou and Stavropoulos [91], who focused mainly on the energy use of the gaseous products and the processing of char in activated carbon with a surface area of 1100 m^2^/g.

### 4.3. Liquid–Solid Pyrolysis

Liquid–solid pyrolysis, otherwise known as “thermolysis”, involves thermally degrading whole tyres immersed in a solvent medium at 360–380 °C in an inert atmosphere of nitrogen. The solvent is a heavy oil which dissolves the oligomers formed during thermal devulcanization and depolymerization. The decomposition products in this liquid mixture at room temperature appear as an oil-carbon-black suspension. This thermolysis, applicable to both sulfur-vulcanized and peroxide-crosslinked rubbers, was explored for tyres by Bouvier et al. [61,92]. An aromatic oil has proved useful for the thermal degradation of styrene-butadiene rubber, but a paraffin oil gives better results for ethylene-propylene-diene rubber. Overall, the solvent oil used should be thermally stable, chemically compatible with the degradation products and inexpensive, and its vapor pressure must be low so that thermolysis can proceed at a pressure close to atmospheric [61].

Liquid–solid pyrolysis in a nitrogen atmosphere can also be maintained in the temperature range *t* = 260–430 °C in the presence of a hydrogen donor such as tetralin. The introduced hydrogen can attach to the free radicals released during the thermal degradation of rubber block macromolecules, thereby inhibiting the secondary agglomeration and recombination reactions which lead to coking. The studies described in [69,91] yielded a gas fraction (C_1_-C_6_ olefins and paraffins), a liquid fraction (hydrocarbons with a molecular weight of 118–300 g/mol) and ca 34% char less than in conventional pyrolysis. The advantage of this type of pyrolysis is the higher proportion of the liquid fraction and the lower process temperature.

A modification of liquid–solid tyre pyrolysis is to replace the oil solvent with water [48,49,50,51] or supercritical carbon dioxide [52,53]. This hydrous pyrolysis performed at high temperatures (250–400 °C) and pressures (4–22 MPa), sometimes self-generated by gaseous products [51], yields the following fractions: water, gas, oil (ca 70%) and solid residue (ca 30%). In water, the thermal degradation of rubber gives greater yields because its supercritical state temperature and dielectric constant are higher than those of CO_2_ (Figure 3), and also because of its nucleophilic character.

When the pyrolysis is carried out in supercritical CO_2_, the temperature is lower and so is the degree of degradation of the tyre, but the proportion of liquid hydrocarbons to char is still better than in the case of vacuum pyrolysis or when the process is carried out at atmospheric pressure [93].

### 4.4. Catalytic Pyrolysis

Polymeric organic materials in tyres decompose faster in the presence of a catalyst than during thermal degradation. The use of a catalyst in water tyre pyrolysis enables heavy hydrocarbons to be cracked into lower molecular pyrolytic oils. A wide range of catalysts (Friedel–Crafts catalyst, zeolites Y, ZSM-5 ([62,63,94]) and HY and HZSM-5 ([67]), MgO/CaCO_3_ ([65]), Na_2_CO_3_/NaOH ([23]), acidic solids, and bifunctional solids) are employed for this purpose [62,63,64,95,96]. Larsen tested the catalytic cracking of waste rubber (scrap tire) by using molten salts of ZnCl_2_, SnCl_2_, and SbI_3_ (Lewis catalysts) at 380–500 °C [97]. The yields of oil (38–78%), gas (10–17%) and solid residues (45–49%) were similar to those obtained by thermal decomposition [51].

The influence of zeolite HY and HZSM-5 in tyre pyrolysis on the yield of limonene, among other compounds, is described in [67]. Type Y and ZSM-5 zeolites as well as acidic crystalline aluminosilicate with a high ion exchange capacity, facilitate the cracking and isomerization of C–C bonds in hydrocarbons, and are therefore commonly used in these reactions.

Tyre pyrolysis in a fixed bed reactor with a type Y catalyst at *t* = ca 500 °C increases the gas yield but reduces the oil yield compared with conventional pyrolysis. However, this oil contains a significantly higher concentration of aromatic compounds (24% toluene, 5% benzene and ca 27% xylenes). An even higher percentage of aromatic compounds in oil was obtained with a ZSM-5 catalyst: this is because this zeolite has a higher Si: Al ratio, a lower acidity and smaller pores than zeolite Y [63,71,98].

## 5. Analysis of the Literature with Respect to Limonene Yield

D-limonene on an industrial scale is extracted from the citrus rind, especially in Florida (USA) and Brazil. The oil (with a limonene) is extracted is from the waste part of fruits, which are separated after the juice production. D- limonene is used in cosmetics and cleaning products, and demand on this component is still growing, with projections showing that it could be as much as 70%, increasing from 2011 to 2021.

In this article the alternative way of limonene potential production is reviewed; pyrolysis of waste tyres could be a source of D- limonene, which is a component of oil fraction. This method could be a complementation of currently used technologies and, what is more, will be of economic value in pyrolysis in the used tyres industry.

Due to the restricted scope of this paper, the drawings of reactors in the cited works are omitted here, especially that they can be accessed in [1]. In addition, Table 1 summarizes the reasoning underlying the analysis of the optimal parameters of limonene production (temperature, pressure, heating rate, residence time of gaseous products in the reactor, particle size of rubber waste, etc.) for all the results of the various authors cited in this study.

The more detailed analysis embraces just a few of the most characteristic publications relating to limonene production.

### 5.1. The Pyrocycling^TM^ Technology as Exemplifying Limonene Production

The semi-continuous vacuum pyrolysis of shredded tyres, known as Pyrocycling™, was developed by Pyrovac. The process, installation and relevant technological procedures are described in [56]. This paper also discusses the optimum operating conditions for the production of *dl*-limonene and its separation from the pyrolysis oil. The Pyrocycling ^TM^ pilot plant is illustrated diagrammatically in [1,56,66,70,119]

The main element of the large-scale experimental vacuum pyrolysis unit is a 3-m long semi-continuous reactor, with a diameter of 0.6 m. Two horizontal plates, each 0.35 m wide, are placed inside the tube of this reactor, one above the other. Both are heated by eutectic molten salts, circulating countercurrently with the feedstock through the tubes. Shredded tyres of granule volume <3.8 cm^3^ or 2.7 cm^3^ (experiment DO14) are fed at a rate between 21 and 42 kg/h and conveyed over both heating plates while being agitated using a novel patented device [56]. At *t* = 438–534 °C and at absolute pressure *p* < 12 kPa, the tyres are pyrolyzed into gas, gaseous light and heavy hydrocarbon vapors, as well as a solid fraction, mainly char, containing steel cords and a small amount of ash.

The vapors and gases are sucked from the reactor and flow through two packed towers indirectly cooled with tap water. Heavy oil, condensed in the first column, and light oil, condensed in the second one, are collected separately in tanks. A ring liquid vacuum pump removes flammable gas from the installation and compresses it in a storage tank. A more detailed description of the Pyrocycling™ installation, tested over several years by Mirmirana, is given in his dissertation [119].

The technological parameters of the vacuum pyrolysis of tyres and polyisoprene are summarized in Table 1. Car tyres (D014) and truck tyres (H018, H036 and H045) were processed in a semi-continuous pilot plant reactor, whereas truck tyres (A120, A121 and A122) and pure polyisoprene (G45) were pyrolyzed in periodic mode in two small batch reactors with respective capacities of 1 dm^3^ and 15 dm^3^ [56,58,78].

The ***dl***-limonene-rich oil fractions D014, H018 and H036 were distilled in a 0.75 m long and 0.945 m diameter glass column of capacity 0.3 m^3^, packed with a metallic material. The recovered naphtha fractions were then once more batchwise distilled, but in a 5 × 10^−3^ m^3^ capacity column with the following results for the boiling point *t*_b_ = 175–176 °C: 92% of the ***dl***-limonene concentration (D014), 50% (H018) and 63% (H036)

Analysis of the results of the Pyrocycling™ pyrolysis technology shows that the highest limonene yield was obtained from natural rubber of truck tyres in procedure H045 (3.6%) (Table 1), or when the charge was polyisoprene G45 (9.8%).

### 5.2. Pressure Effect on Vacuum Pyrolysis of Polyisoprene and Waste Polyisoprene Tyres

The vacuum pyrolysis technology described in [58] is a development of the technology described above [56]. The pyrolysis was carried out in the same (G45) small vacuum pyrolizer (***V*** = 15 dm^3^). The charge was natural rubber TTR-20—pure polyisoprene or truck and bus tyres—which contain >50% natural rubber. For example, Goodyear TM 17,343 tyres contain 52.2% of pure natural rubber, 31.0% carbon black, 5.4% butadiene styrene rubber (60% styrene), 4.4% oil (plasticizer), 1.6% ZnO, 1.1% stearic acid, 1.1% sulfur and 3.2% other components [58].

In this vacuum technology, milled tyre granules of medium size (2 cm^3^) were heated at a rate of 15 K/min from *t* = 25 °C to *t* = 500 °C. The pyrolysis was performed in a protective atmosphere of pyrolysis gas, which was sucked from the pyrolizer under vacuum (*p* = 0.0008 MPa; 0.8 kPa) in the experiment designated G42 for polyisoprene. The remaining experiments were the following: G47 (tyre*, p* = 0.0064 MPa), G58 (polyisoprene, *p* = 0.028 MPa) and G45 (isoprene). The vacuum in the plant was produced by a vacuum pump. The pyrolytic oil was condensed in three series of radiator coolers at temperatures from *t* = −15 °C to *t* = −78 °C. The blend of the three oil fractions was distilled at a temperature of *t* = 240 °C at atmospheric pressure, according to ASTM procedure D86 [58]. Two fractions were obtained as a result: petrol and oil.

The largest proportion of ***dl***-limonene was contained in the products of the liquid vacuum pyrolysis of polyisoprene. The concentrations of other products, mainly olefins and low molecular weight aromatic hydrocarbons, increased with increasing pyrolysis temperature. The mechanisms of the thermal depolymerization of polyisoprene into dimers and isoprene monomers and the formation from them of limonene and isoprene are described by the Diels–Alder reaction. Those papers also indicate how limonene or other compounds decompose at elevated temperature or in the presence of oxidants [120,121,122].

Table 1 shows the effect of pressure on the proportions of the separate pyrolysis products, particularly the yield of limonene in the vacuum pyrolysis of pure polyisoprene (natural rubber) or the products in which it is dominant.

The yield of the naphtha fraction obtained from pure polyisoprene does not depend on the pressure of its pyrolysis. In the case of polyisoprene rubber pyrolysis, however, this yield decreases with increasing pressure, although the composition of the petroleum fraction obtained at *p* = 0.8 kPa and 28.0 kPa is similar, as demonstrated by the results of the tentative characterization of the pyrolytic naphtha fraction at the initial boiling point IBP 204 °C from the pyrolysis of polyisoprene (P) and polyisoprene rubber (PR) at various pressures at *t* = 500 °C [58]. The results of GC-MS analysis for the retention time *t*_R_ = 22.16 [min] corresponding to *dl*-limonene, expressing the yield as a percentage area, are as follows: 54.64 for polyisoprene (P) and *p* = 0.8 kPa and for polyisoprene rubber (PR) 31.4 (*p* = 0.8 kPa) and 31.22 (*p* = 6.4 kPa).

The light fraction of oil-derived vacuum pyrolytic contains significant amounts of valuable ***dl***-limonene. The concentration of this compound increased with decreasing pyrolysis pressure. The rest of this fraction consists mainly of aromatics and branched olefins.

### 5.3. Temperature Effect on Vacuum Pyrolysis of Crushed or Whole Tyres

This section discusses further research by Roy, Chaal and Darmstadt, who this time carried out on a more in-depth study of the effect of temperature on the pyrolysis of different types of tyres and of varying degrees of fragmentation. The research was carried out in a pyrolizer previously described by Pyrocycling™ technology [56,58,59,78].

Larger pieces of chipped tyres and whole tyres were thermally degraded in a periodic pyrolizer. After being loaded, it was closed and heated at a rate of 10 K/min. In experiment H20, the raw material consisted of comminuted car tyre fragments (size 100 × 120 mm, total weight 158 kg); in H21 it also comprised car tyres, but with a silica filler (total weight 80 kg); while in H22 it was crushed truck and bus tyres (ca 100 × 120 mm, 180 kg).

Test results [123] from experiment H18 were obtained on a ca 20 × 30 mm granulate obtained from car tyres that were degraded in a continuous, electrically heated pyrolizer.

Vacuum pyrolysis yields of oil from different feedstocks, e.g., car or truck tyres with or without a silica filler, and the mass balances for the experiments were: 56% (H18, *p* = 10 kPa, *t* = 550 °C), 45% (H20, *p* = 7 kPa, *t* = 520 °C), 47% (H21, *p* = 7 kPa, *t* = 500 °C) and 43% (H22, *p* = 6 kPa, *t* = 483 °C) [59]. These results are not listed in Table 1 because they are not directly related to limonene yield.

The yields of oil summarized above are consistent with previous work [60] and studies of the influence of pressure and temperature [78]; they confirm that the oil yield is higher from vacuum than from atmospheric pyrolysis, whereas the char and gas yields are smaller [106] and, in addition, undesirable reactions of the secondary decomposition of valuable compounds such as *dl*-limonene are limited.

The calorific value of the oil fraction (ca 44 MJ/kg) is higher than that of the tyre itself (33 MJ/kg) and the char (28 MJ/kg of bituminous coal or 30 MJ/kg of technical soot), which together with the low ash (<0.005%) and sulfur contents (0.8–1.5%) allows this fraction to be used as fuel or diesel oil [124]. However, limonene is more expensive than diesel fuel, so combustion of the latter without prior limonene separation is unprofitable.

The pyrolytic oil can be distilled into different fractions. In [59] the following, sometimes overlapping, oil fractions were studied: IBP −204 °C, >204 °C, 240–450 °C, >350 °C and >400 °C.

The fraction with IBP = 204 °C, distilled up to 160 °C, is similar to the light naphtha fraction produced from petroleum. Chromatographic analysis showed this oil fraction to consist of 45% aromatics, 22% olefins, 15% isoparaffins, 1% n-paraffins, 7% naphthenes and 10% high molecular-weight hydrocarbons and heterocyclic compounds [59,70]. Only this fraction, which constitutes ca 26.8% of the pyrolysis oil yield [123], contains 31.2% to 54.6% [58]) of limonene.

### 5.4. Pyrolysis technology in PDU

This is another vacuum pyrolysis technology, studied over a wide range of temperatures by Pakdel, Roy and colleagues [57]. They used the PDU (Pyrolysis Development Unit) installation as described in Aubin’s doctoral dissertation [125].

The main component of this pilot installation is a semi-continuous vertical cylindrical pyrolizer (diameter *D* = 0.7, height *H* = 2 m), with six heating zones that stabilize the horizontal layers of granules for specific temperatures: *t* = 226 (highest layer), 295, 366, 404, 450 and 510 °C. The maximum temperature and total pressure in the reactor were 510 °C (at the bottom of the pyrolizer) and 1 kPa, respectively.

Shredded cross-ply tyres (6–12 mm Tyler sieves) were dispensed and moved by gravity to the pyrolizer through a double sluice. The feeding rate was 3.5 kg/h. The solid fraction was also drained from the bottom through a double sluice.

The organic vapors (hydrocarbon gases and liquids) were sucked out of the reactor chamber by a mechanical vacuum pump and condensed in a series of six condensers (H-I, H-II … H-VI). The condensers were installed in parallel at the reactor outlets, corresponding to the six reactor hearths. The remaining non-condensed vapors and gases were collected in a train of receivers in the second stage of condensation C-I, C-II ... C-IV. A mixture of the liquid fractions from the first and second condensing units was distilled under atmospheric pressure up to 204 °C, to separate the naphtha fraction containing limonene. This yield of this fraction represents 26.8% of the total pyrolysis oil.

On average, the process yielded 55% oil, 25% carbon black, 9% steel, 5% fiber residues and 6% gas. Dipentene (***ld***-Limonene) made up 15% of the naphtha fraction, or 2.2% of the as-received tyres. Limonene was definitively identified by co-injection of an authentic sample of dipentene (from Aldrich) and GC/FTIR analysis. Polarimetric analysis of the purified >80% limonene sample also confirmed its racemic structure [57].

Vacuum pyrolysis of spent tyres produces ca 55% of oil with a molecular weight range from a nominal 50 to 120 [55] and typically contains 20–25% of the naphtha fraction with a boiling point <200 °C. The naphtha fraction typically contains 20–25% ***dl***- limonene [56,126].

### 5.5. The Effects of Additives on Vacuum Pyrolysis

The effects of temperature and basic additives (anhydrous Na_2_CO_3_ and NaOH) on the vacuum pyrolysis of a worn tyre granulate are discussed in [23]. This laboratory study was performed in a cylindrical, stainless steel pyrolysis reactor with an inner diameter of 32 mm and a height of 120 mm. Several slanting, stainless steel plates were fixed in the reactor to intensify heat transfer from the electrical furnace.

Periodically, 100 g of tyre granules mixed manually with 3.0 g of additives were loaded into the reactor, after which this was sealed. The air inside the reactor was evacuated by a vacuum pump (3.5–10 kPa), after which heating commenced to the desired temperature (450–600 °C) at an average rate of 20 K/min. The vapors were condensed in an outer water cooler. The uncondensed vapors were condensed further by four cold traps containing water–ice–sodium chloride.

The yields of oil and ***dl***-limonene as a function of different vacuum pyrolysis temperatures (*t* = 450, 500 and 550 °C) maintained at *p* = 3.5–4.0 and 10 kPa with and without additives (Na_2_CO_3_ and NaOH) are listed in Table 1. These results indicate that the addition of NaOH could lower pyrolysis temperatures and also increase the oil yield to 49.7% at 480 °C. The addition of Na_2_CO_3_ did not influence the pyrolysis process. If these catalysts are added to tyre granules with the same pyrolysis parameters (500 °C and 3.5–4 kPa), the limonene yield in the oil obtained increases slightly from 11.73% to 11.95% with NaOH and to 12.39% with Na_2_CO_3_.

### 5.6. Influence of the Technological Pyrolysis Parameters of the Inert Gas on Limonene Yield

The heat carrier in this semi-technical technology is heated nitrogen, which also serves as a protective gas. This flushes the individual tyre granules and penetrates the entire bed, thus ensuring uninterrupted heating without mechanical mixing, as well as gasification or pyrolytic tyre decomposition. Another purpose of the inert gas is to flush out the oxygen from the reactor at the beginning of the pyrolysis process and the thermal decomposition products during its course.

The aim of studies [25,103,104] was to investigate the temperature, particle size, flow rate and inert gas flow rate effects on pyrolysis, in order to maximize the limonene yield.

The set-up consists of the following major components: a 0.1 m diameter reactor chamber with fixed-bed fire-tube heating and thermally isolated with asbestos, a gravity reactor feeder, two ice-cooled condensers with a glass liquid collector at the bottom, a *N*_2_ gas cylinder with pressure regulator and gas flow meter, a *N*_2_ gas pre-heater with LPG burner, air compressor, char collecting bag and thermocouples (chromel-alumel) with temperature controller. A stainless-steel distributor plate with 150 holes of 0.003 m diameter was fitted at a distance of 0.03 m from the bottom of the reactor to support the feedstock. For heating the feedstock, eight equally spaced stainless-steel fiber-tubes of 0.01 m in diameter containing an insulated electric coil with a total capacity 1.6 kW were fixed inside the reactor.

The pressure in the reactor was slightly higher than atmospheric. The inert gas, at room temperature at the reactor input, had a volumetric flow rate of from 2, 4 to 8 L/min; this flow rate increased the reaction temperature in the reactor by 2.5 times. In accordance with the gas laws (Boyle’s and Charles’ combined law), this is due to the higher reactor, which for pyrolysis is about 475 °C, as a result of which gaseous and volatile pyrolysis products form. The sample was heated at heating rates of 5–20 K/min to the desired temperature (375, 425, 475, 525, 575 °C). Pyrolysis was continued until the inflow vapor product to the receivers ceased to be visible; the process thus lasted about 50 min. The volatile fractions, condensed in coolers, were collected in vessels until these were filled. Therefore, the results of composition analysis and performance were not affected by the presence of atmospheric air. After weighing, the heavy fraction was centrifuged for 15 min at 3000 rpm to remove solid impurities and inclusions. On leaving the cooler, the residual gas was burned in a torch.

The feed materials used in a pyrolytic study consisted of chopped, heavy automotive tyre granules of size ***v***_I_ = 8 × 1 × 0.25 mm = 2 mm^3^, ***v***_II_ = 8 × 1 × 0.5 mm = 4 mm^3^, ***v***_III_ = 8 × 1 × 1 mm = 8 mm^3^ and ***v***_IV_ = 8 × 1 × 1.5 mm = 12 mm^3^. The sample contained 55% NR (natural rubber), 35% SBR (styrene-butadiene rubber), 10% BR (butadiene rubber) and textile netting, but no steel wire or cords.

The effect of heating rate was studied with the aim of finding a set of technological parameters for obtaining the maximum amount of oil; these optimal values were found to be *t*_opt_ = 475 °C, ***v***_opt_ = 4 cm^3^, *RH*_opt_ = 15 K/min and the flow of nitrogen *V* = 8 dm^3^/min [25].

The results presented in papers [25,103,104] and compared in Table 1, as well as other researchers ([89,101,106]), indicate, among other things, that the amount of limonene decreases with increasing pyrolysis temperature. This is the effect of the secondary thermal decomposition of limonene, described in Figure 5.

### 5.7. Tabular Summary of the Remaining Results

All the results, both those discussed above and those omitted in the description, are listed in the same way in Table 1, i.e., the dependence of the mass yield of pyrolysis oil and limonene as a function of temperature, conditions for the pyrolysis of used tyres (vacuum, catalytic, in the presence of an inert gas), type of installation and scale of the process.

Scale: L-laboratory plant, LSE-large scale experimental, T-technical plant, P-pilot plant (l) - ***l***-limonene, (d)- ***d***-limonene

The results from Table 1 below are presented in the form of graphs; it should be mentioned that not all data was included in the graph, the content of limonene which was given in publications as the area of the peak marked on the chromatogram was insufficient to be calculated into% and to compare with other results.

**Figure 6:**
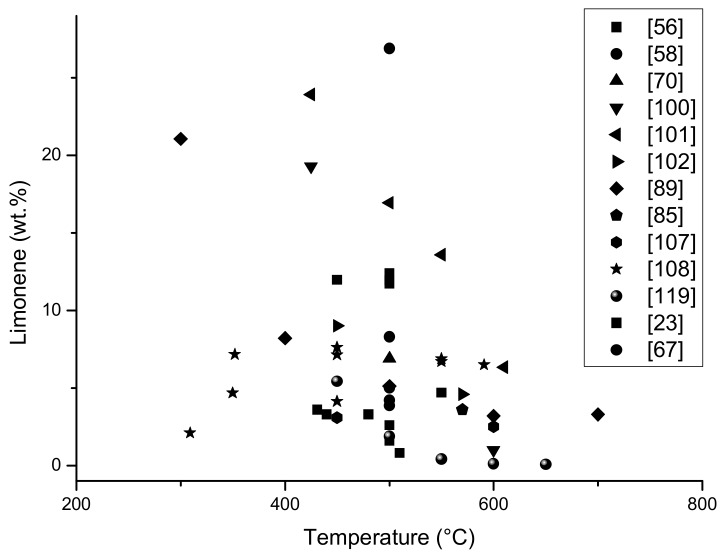
An amount of limonene (%) in oil fraction dependence on the temperature of different pyrolysis process (based on Table 1).

**Figure 7:**
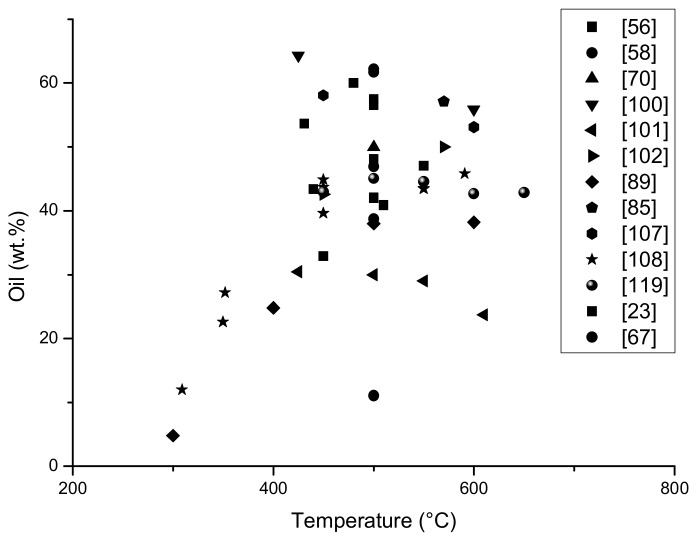
The yield of oil fraction (%) dependence on the temperature of the different pyrolysis process (based on Table 1).

On the graphs (Figure 6 and Figure 7) it is presented the amount of limonene obtained depending on the temperature of the pyrolysis process and the particle size of the feed material. In addition, the amount of oil fraction obtained has been demonstrated depending on the process temperature, since limonene is part of the oil, it can be concluded that as the amount of oil fraction increases, the amount of limonene obtained in the process also increases.

On Figure 6, the limonene yields, obtained in different pyrolysis processes, are presented. Between 400–600 °C, the highest amount of limonene was obtained. In [89], authors determined about 20% of limonene in oil fraction in 300 °C, and this is a surprisingly good result, especially in the case that in 300 °C tyres, pyrolysis could be unfinished. It needs to be highlighted that all results came from laboratory equipment, where the samples (raw materials) are a small amount (2 g) and only from waste tyres. The pure polyisoprene sample wasn’t included on the graph, but the various type of raw materials and various polypropylene content influence on the limonene yield. Above 600 °C, less limonene was obtained, which is associated with its degradation at higher temperatures.

The content of the liquid fraction obtained in pyrolysis is the highest in the temperature range 420 to 600 °C (Figure 7). The highest yield of oil is about 50–60%, the least liquid fraction was obtained at a temperature below 400 °C it was even below 20%. It is worth noticing that the table shows the pyrolysis balance only of processes in which the content of limonene in the liquid fraction was determined, so it can be assumed that the process was focused on the highest pyrolytic oil yield.

**Figure 8:**
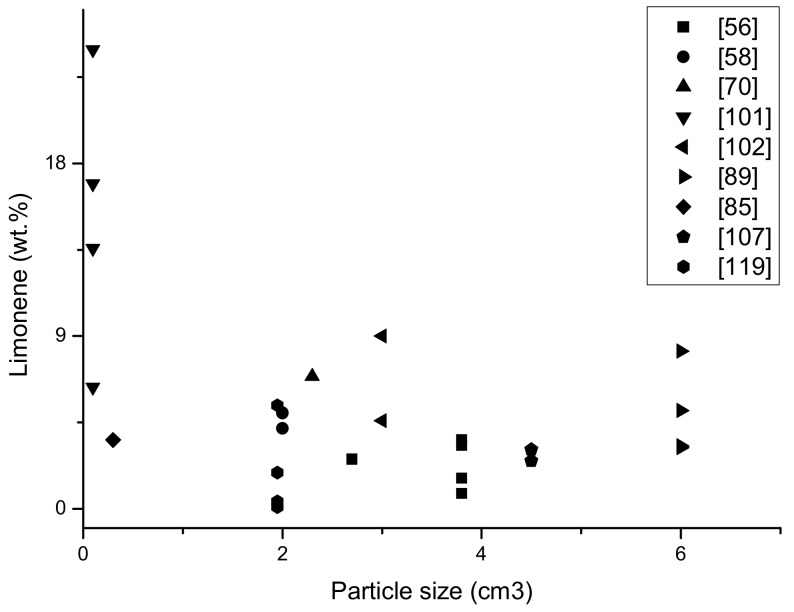
The yield of limonene (%) in oil fraction dependence on the particle size of waste tyres of different pyrolysis process (based on Table 1).

The last graph (Figure 8) shows the dependence of the comminution of the input material on obtaining limonene in pyrolytic oil (%). The data from Table 1 was take into account; in the case of a few publications there were not cubic values, but only the surface of pieces. It was assumed that with high fragmentation material (about 1 mm), the third dimension was adopted for 1 mm (the same as other dimensions), with the values given in cm; the thicknesses were calculated as 0.5 cm so that they could be compared each other. The highest amount of limonene was obtained in the samples with the largest fragmentation, which is associated with better heat reaching in a single particle and more effective heating, and thus reaching higher temperatures in a single tire particle and more efficient tire distribution. In the samples with less fragmentation, comparable amounts of limonene were determined. A pyrolysis process was performed on a laboratory scale, with an influence similar to the size of the input material.

When analyzing the results of tire pyrolysis investigations, in terms of the possibility of obtaining limonene from pyrolysis oil, the part of the information about the kinetics of the pyrolysis process, activation energy was presented especially. This information could not be included in Table 1, due to the fact that typically the results of kinetic analysis of the pyrolysis process are obtained on a laboratory scale using: TGA (thermogravimetric analyzer), DSC (differential scanning calorimeter), TGA/DSC or MS (mass spectrometer). While much more research, collected in the Table 1, has been conducted on a larger scale reactor. Therefore, we decided to put the results of kinetic tests in a separate Table 2.

Analyzing the results from Table 2, it could be observed that only two authors conducted the kinetics parameters of isoprene and ***dl***-limonene formation during waste tyres pyrolysis [129,130]. In other research, the activation energy values for various pyrolysis stages or different products as a function of tire type and composition, heating rate, pressure and catalyst presence, were compared. Therefore, the conclusions based on only two results would be insufficient; in this case, it is needed to take this into consideration in the further kinetic studies of the obtaining of limonene from tire pyrolysis.

## 6. Conclusions

The most important conclusions to be drawn from the analysis of the above pyrolysis results are that:

- the highest quantity of *dl*-limonene (3.6%) was obtained from truck tyres using procedure H045, described in Table 1;
- at *t* > 500 °C, limonene decomposes to trimethylene benzene, *m*-cymene and indane, the boiling points of which are close to that of ***dl***-limonene; the pyrolysis temperature should therefore not exceed 500 °C;
- the ***dl***-limonene yield increases with decreasing pyrolysis pressure. Earlier studies by Roy [58] showed that reducing the pyrolysis pressure from 0.028 MPa to 0.008 MPa resulted in a 30–40% increase in the amount of ***dl***-limonene in the oil;
- the disruption of heat and mass exchange (under- or overheating) during pyrolysis inhibits the formation of ***dl***-limonene or promotes its secondary degradation;
- the dimensions of the material to be pyrolyzed also has a large impact: the smaller the size of the rubber granulate from the disintegrated tyres; the more oil and limonene are obtained.

The conditions that facilitate increasing limonene yields, i.e., low temperature and pressure, fast heating rate and short residence time of volatile products in the reaction environment—are best obtained in the following types of pyrolizers (in descending order of efficiency): vacuum mechanical, fixed bed, inert gas (gas-solid pyrolysis) and catalyst, fluidized bed and mechanical pyrolizers.

In general, the higher the proportion and oil yield in a tyre pyrolysis product, the potentially greater the quantity of limonene obtained. Unfortunately, we cannot confirm this for all the publications on tyre pyrolysis that we analyzed, because many of their authors did not carry out a detailed analysis of the oil, treating it as liquid fuel and testing it only in this respect (viscosity, density, calorific value, etc.). Those authors who did analyze the presence of limonene in oil determined its chromatographic amounts as a mixture of ***dl***-limonene enantiomers. However, its mass yields were related to the mass of tyres or the amount of oil obtained; they did not always state whether it was all oil or just one of the fractions. We have tried to give this information in Table 1, but it was not always given by the authors.

The pyrolytic liquid fraction is a complicated mixture of aromatic compounds, which were formed in the degradation of polymers. The separation of limonene and purification, as a valuable chemical, from TPO is a complicated process. The main method used in much research is the distillation process, which could be one of the methods of enriching limonene in oil fraction. The limonene (boiling point 176 °C) could be separate with the light naphtha fraction (boiling point below 200 °C), which contain the majority of limonene from all liquid fraction. The purification or isolation of limonene from this fraction isn’t simple. On the laboratory scale, it was conducted by Pakdel et al. [56,57], and they received a 95% of pure dipentene, but it is not possible to completely separate derivatives of limonene with similar boiling points (such an indane). This two steps method was used by Pakdel et al.: distillation process and the solid-liquid chromatography, but separation limonene on semi-technical or technical scale is still a scientific challenge. Williams and Brindle [85] proposed selective condensation of the reactor outlet. Another way to induce limonene separation is via the receipt of derivatives or using ion chromatography.

One should also be aware that the pathway from the analytical (chromatographic) amount of ***dl***-limonene in oil to the actual amount of ***d***-limonene, ***l*-**limonene or ***dl***-limonene obtained technologically from waste tyres is still a long one.

## Figures and Tables

**Table 1:** Literature data parameters of batch bed reactors for the pyrolysis of spent tyres and rubber materials.

Ref.	Scale	Oil%	*T* °C/*p* kPa	Concentrations of Limonene in Pyrolytic Oils %		Type of Tyre/Material Particle Size	Type of Reactor
**Vacuum**							
[56]	LSE	57.5 56.5 40.9 53.7 60.0 43.4 90.3	500/12 500/13 510/10 431/12 480/13 440/13 500/28	2.6 (D014) 1.6 (H018) 0.8 (H036) 3.6 (H045) 3.3 (A120) 3.3 (A121) 9.8 (G45)	car tyres track tyres -||- -||- -||- -||- polyisoprene	granules > 2.7 cm^3^ <3.8 cm^3^ -||- -||- -||- -||- 2 cm^3^	semi-continuous horizontal, pilot -||- -||- batch, 1 dm^3^ -||- batch, 15 dm^3^
[57]	P	55.0	226→510/1.0	14.92		shredded cross-ply tyres (6–12 mm)	semi-continuous gravitational batch transport
[58]	T	97.3 90.3 62.2 61.7	500/0.8 500/28.0 500/0.8 500/6.4	16.6 11.9 5.0 4.2		pure polyisoprene 2 cm^3^ polyisoprene rubber 2 cm^3^	batch reactor V = 15,000 cm^3^
[70]	P	50	500/13	6.9		cylindrical particles *h* =12 mm; *d* = 6 mm	semi-continuous rake conveyor pyrolizer
**Gas–Solid**							
[99]	L	64.3 55.9	425 600	19.3 >1		tyres 3 g/min (35 g of sand)	fluidized bed CSBR (conical spouted bed reactor)
[100]	L	30.48 ca.30 ca.29 23.70	425 500 550 610	23.93 16.94 13.59 6.34		0.63–1 mm (2 g of sample)	fluidized bed CSBR (conical spouted bed reactor)
[101]	L	42.6 50.0	450 570	9.0 4.6		1–3 cm^2^ pieces	fixed bed
[102]	L	50.5 41.3 50.0 48.7 46.3	500 500 500 (RT = 1 s) 500 (RT = 3 s) 500 (RT = 5 s)	23.9% peak area in oil		size 0.32 mm, size 0.8 mm No data No data	fluidized bed (circulating)
[103]	L	37.8 10.9 <0.01 25.7 13.1 <0.01	450 750 1000 450 750 1000	6904 mg/kg tyre 165 mg/kg ≈ - 4138 mg/kg tyre 1045 mg/kg -	gas N_2_ 1.5 L/min - N_2_ + 10% O_2_ ≈ 1.5 L/min -	particle size 4 mm	fixed bed
[25]	L	52 55 51 49	425 475 525 575	50.8% 50.86 24.84 21.24	area (sum of peak for *t*_R_ = 30.71, 30.90, 30.97 and 31.03) in pyrolysis oil	particle size 4 cm^3^ (optimal from the viewpoint of oil yield)	fixed bed
[89]	L	4.8 24.8 38.0 38.2 38.5	300 400 500 600 700	21.07% 8.22 5.12 3.19 3.29	limonene in oil -||- -||- -||- -||-	particle size 2 × 3 cm	fixed bed
[85]	L	57.1	570	3.6% in oil in oil fraction: <100 °C: 1.5%/imps 6.8% 100–150 °C: 1.1%/imps 2.9% 150–200 °C: 0.7%/imps 5.9% >250 °C: 0.5%/imps 5.6%		particle size 1.0–4.0 mm	fixed bed (with and without packing glass impingers—imps.)
[104]	L	48.5 ± 2 51 ± 1.5 47 ± 1.5 43 ± 2.1	425 475 525 575	11.1% peak area in oil		particle feed size 4 cm^3^	fixed bed
[105]	L	46 49 55	475 475 475	10.95% peak area in oil 29.54 -||- 50.86 -||-		bicycle 4 cm^3^, motorcycle -||-, truck -||-	fixed bed
[87]		34–46	500–550	3–4% peak area in oil		12 kg of particle size 2–6 mm	fixed bed
[106]		58.15 3.1	450 600	3.1 2.5		3 kg of particle size 3 × 1.5 cm	fixed bed
[107]	L	27.20 43.50 22.59 43.43 43.70 11.97 45.84 39.60 44.90	350 550 350 550 450 309 591 450 450	7.17 6.88 4.67 6.70 7.14 2.10 6.49 4.14 7.62	(5 K/min) (5 K/min) (5 K/min) (25 K/min) (15 K/min) (15 K/min) (15 K/min) (0.86 K/min) (29.14 K/min)	tyre	fixed bed
[108]	L	55.0	850	21.58%	(5 K/min)	tyre crumbs	fixed bed
[108]	L	25.0	850	5.12%	(5 K/min)	tyre crumbs	rotary oven
[109]	L	58.4 58.2 54.0	425 475 575	21.82 24.29 8.29 (dl limonene)		waste truck tire 2.8–3.3 mm	CSBR
[110]	L	38.29 30.89 30.48 29.78	500 600 700 800	6.65 3.75 4.18 4.16		tyre	fixed bed
[111]	L	2.5 36.6 3.6 32.8 4.0 32.3 4.8 30.8 5.0 30.0	340 511 338 509 340 509 339 514 336 509	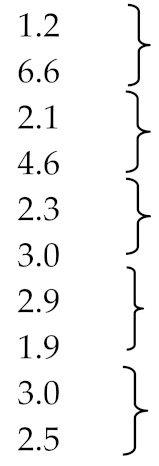	one experiment one experiment one experiment one experiment one experiment	tyre (two stage pyrolysis)	auger fluidized bed auger fluidized bed auger fluidized bed auger fluidized bed auger fluidized bed
[112]	L	1.6 48.0 3.5 44.6 5.0 42.1	229 506 334 511 454 516	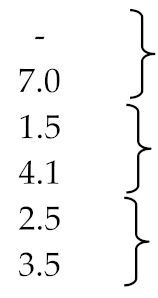	one experiment one experiment one experiment	tyre (two stage pyrolysis)	auger fluidized bed
[113]	L	40.0	500	6.02%		truck tire	microwave- induced
[114]	L	51.0 45.5 63.5	650 750 750	11.11 21.22 12.75	(20 K/min)	light tyres medium tyres heavy tyres	batch pyrolysis reactor
[115]	L	46.6 50.0 58.2	475	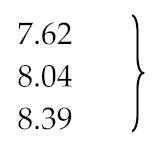	Tube and shell condenser		fixed bed bubbling fluidized bed CSBR
[115]	L	49.2 55.9	475	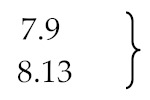	Quenching condenser		fixed bed bubbling fluidized bed
[116]	L	23.4	9 W/g 15 W/g 24 W/g	9.3 9.83 9.16		tyre (d = 0.6 mm)	microwave oven
[117]	L	36 44 45	10 min 20 min 30 min	8.61 9.92 9.83		tyre	microwave oven 0–900 W
**Without inert gas**							
[118]	P	43.0 45.1 44.6 42.7 42.9	450 500 550 600 650	5.440 1.883 0.419 0.122 0.070		shredded scrap tyre with particle sizes of 13–15 mm	mechanical (continuous rotary kiln)
**Vacuum and catalyst**							
[23]	L	32.9 42.1 47.1 - 48.1 42.0	450/3.5–4 500/3.5–4 550/3.5–4 500/10 500/3.5–4 500/3.5–4	11.97 11.73 4.7 2 7.8 11.95 12.39	without catalyst -||- -||- -||- with NaOH with Na_2_CO_3_	100 g tyre granules with 3 g Na_2_CO_3_ or NaOH	fixed bed
**Catalyst and inert gas**							
[67]		46.97 38.76 11.09	500 500 500	1.27 (l)/25.53 (d) 0.62 (l)/7.72 (d) 0.0 (l)/3.86 (d)	without HZSM-5 HY	granulate of used tyres	conical spouted bed
[95]	L	57.9 79.8 35.5 46.1	380 480 380 480	108.8 mg/g_sample_ 120.5 -||- 0.9 -||- 2.2 -||-	without Y-zeolite with Y-zeolite	pieces of cut rubber gloves measuring 1 cm^2^,	fixed bed 200 mL/min N_2_ flow
[62]	L	55.8 ca 48 ca 49 ca 50 ca 45 ca 45 ca 47	500 500 500 500 500 500 500	3.6 ≈0.05 CBV-400 ≈0.04 CBV-780 ≈2.0 ZSM-5 ≈0.01 CBV-400 ≈0.02 CBV-780 ≈0.2 ZSM-5	without catalyst 0.25 ratio -||- -||- 0.5 ratio -||- -||-	shredded tyre of size 1.0–1.4 mm, catalyst: 1 mm in diameter, 5 mm in length	fixed bed 100 mm diameter 150 mm high, 200 g sample, the inert gas was nitrogen

**Table 2:** Comparison of the activation energy of the pyrolysis process of rubber and tyres.

Ref.	Activation Energy [kJ/mol]	Carrier Gas/Pressure [bar]	Temperature [°C]	Conditions	Material
[121]	225 ± 5	N_2_	270–320	Isothermal	Guayule Rubber in the presence of stearic, oleic, linoleic, and linolenic acid
	167 ± 5	air	200–265	Isothermal	
	90	air	57–125	Isothermal	
	239	N_2_ and air		Dynamic	
[88] [127] [128]	66.8/44.8/32.9	N_2_	Regions: (1) 150–250 (2) 200–335 (3) 320–500	5–20 K/min	Shredded automobile tyres
	93.4/78.4/61.1			40–50 K/min	
	52.5/164.5/136.1			10–60 K/min	
	42.0/195.0/204.0			-	
	125.7/178.5/243.7			30 K/min	
[129]	131 for isoprene 67	Ar/10^−6^ Best-fit model	up to 800	up to 100 K/min	Truck tyre sample 0.6–0.8 mm
	115 dl-limonene 93				
	141 for isoprene	Friedman method			
	145 for dl-limonene				
	129 for isoprene	Kissinger method			
	113 for dl-limonene				
[130]	107.6 for isoprene	from Arrhe-nius equation	300–700		Scrap tyre particle size smaller than 0.2 mm.
	96.7 for limonene				
[131]	68.59^a/^69.71^b^	N_2_	up to 700	5 K/min	Scrap tyre and mixture with *Juglans regia* shell.
	73.54^a^/83.44^b^			10 K/min	
	76.56^a^/86.11^b^			15 K/min	
	67.21^a^/75.63^b^			20 K/min	
	Arrhenius (^a^) and Coats–Redfern (^b^) methods				
[132]	125.6 Lump I	He/1	420–450 up to 900	30 K/min	Granulated tyres
	178.7 Lump II				
	244.1 Lump III				
[74] [75]	152.0 (evaporation)	molten lead/0.03	180–480		large tyre particle (SBR)
	215.0 (evaporation)				(BR)
	207.0 (decomposition)				(SBR)
	207.0 (decomposition				(BR)
[133] [134] [30]	69.73 (MLR_1_)	N_2_	Up to 700	2, 5, 10 and 20 K/min	Waste tyre samples of 10, 20 and 30 mg
	118.04 (MLR_2_)				
	128.92 (MLR_3_)				
	Pyrolysis of: MLR_1_—tyre additives, MLR_2_—depolymerized rubbers, MLR_3_—crosslinked/cyclized rubbers				
[135]	147.25		500–600	Isothermal	15 mg samples of HDPE
[136]	50.6 ± 4.9^c^/43.5 ± 3.9^d^	He	503, 625 and 723	Step I	Scrap tyre (1/3 NR, 1/3 SBR and 1/3 carbon black) <0.2 mm
	130.8 ± 13^c^/104.7 ± 9^d^			Step II	
	245.9 ± 19^c^/243.9 ± 21^d^			Step III	
	126.7 ± 12^c^/107.9 ± 8^d^				NR
	201.1 ± 14^c^/219.6 ± 19^d^				SBR
	Pressure (c) 1 bar/(d) 0.25 bar				
[137]	147.64	N_2_	Up to 1000	5, 10, 20 and 30 K/min	Scrap car tyres
	148.06				Scrap truck tyres
[138]	65.42^e^/118.35^f^/108.85^g^	N_2_	30–800	10 K/min	Bicycle/rickshaw tire
	70.45^e^/138.95^f^/105.65^g^			60 K/min	
	79.94^e^/130.25^f^/110.80^g^			10 K/min	Motorcycle tire
	79.94^e^/153.45^f^/99.79^g^			60 K/min	
	74.42^e^/115.87^f^/93.89^g^			10 K/min	Truck tire
	78.19^e^/135.49^f^/95.45^g^			60 K/min	
	Temperature region: (^e^) low (150–350), (^f^) medium (285–450) and (^g^) high (350–500)				
[139]	43 (oil)	N_2_ flow rate 150 mL/min	450–1000	10, 20, 30, 40 and 50 K/min	Raw scrap tire samples from a commercial recycler (∼1 mm)
	207 (NR)				
	152 (SBR)				
	215 (BR)				
[140]	63.08 tyre to gas	N_2_ flow rate 6.6 L/min, sand	425, 500, 550 and 610	Conical spouted bed reactor	Scrap tyre (1/3 natural rubber, 1/3 SBR and 1/3 carbon black)
	40.06 tyre to oil				
	89.26 -||- to aromatics				
[141]	136.1	N_2_	350–500	10 K/min	Tyre powder (40 mesh)
	133.6		370–510	30 K/min	
	107.0		400–540	45 K/min	
	99.1		410–540	60 K/min	
[28]	5.9^h^/559^i^	Activation energy evaluated for thermal (^h^) and catalytic pyrolysis (^i^) with the use of two types of lumping models; discrete and continuous lumping models.			Literature data of thermal [142] and catalytic [63] pyrolysis of scrap tyres were used to verify the calculations
	2.0^h^/6.48^i^				
	0^h^/3.48^i^				
	2.91^h^/12.4^i^				
	–/4.64^i^				

^a^ Arrhenius method; ^b^ Coats–Redfern method; ^c^ 1.0 bar pressure; ^d^ 0.25 bar pressure; ^e^ temperature range: low (150–350); ^f^ temperature range: medium (285–450); ^g^ temperature range: high (350–500); ^h^ thermal pyrolysis; ^i^ catalytic pyrolysis.

## References

[1] Lewandowski W.M., Januszewicz K., Kosakowski W. (2019). Efficiency and proportions of waste tyre pyrolysis products depending on the reactor type—A review. J. Anal. Appl. Pyrolysis.

[2] Elegbede J.A., Elson C.E., Qureshi A., Tanner M.A., Gould M.N. (1984). Inhibition of DMBA-induced mammary cancer by the monoterpene d-limonene. Carcinogenesis.

[3] Elson C.E., Maltzman T.H., Boston J.L., Tanner M.A., Gould M.N. (1988). Anti-carcinogenic activity of d-limonene during the initiation and promotion/progression stages of DMBA-induced rat mammary carcinogenesis. Carcinogenesis.

[4] Bardon S., Foussard V., Fournel S., Loubat A. (2002). Monoterpenes inhibit proliferation of human colon cancer cells by modulating cell cycle-related protein expression. Cancer Lett..

[5] Gupta A., Myrdal P.B. (2004). Development of a perillyl alcohol topical cream formulation. Int. J. Pharm..

[6] Back N., Cohen I., Kritchevsky D., Lajtha A., Paoletti R. (1995). Dietary Phytochemicals in Cances Prevention and Treatment. Advances in Experimental Medicine and Biology.

[7] Trytek M., Paduch R., Fiedurek J., Kandefer-Szerszeń M. (2007). Monoterpeny—Stare zwia̧zki, nowe zastosowania i biotechnologiczne metody ich otrzymywania. Biotechnologia.

[8] Crowell P.L., Chang R.R., Ren Z., Elson C.E., Gould M.N. (1991). Selective inhibition of isoprenylation of 21–26-kDa proteins by the anticarcinogen d-limonene and its metabolites. J. Biol. Chem..

[9] Crowell P.L., Gould M.N. (1994). Chemoprevention and therapy of cancer by d-limonene. Crit. Rev. Oncog..

[10] Ahn K.J., Lee C.K., Choi E.K., Griffin R., Song C.W., Park H.J. (2003). Cytotoxicity of perillyl alcohol against cancer cells is potentiated by hyperthermia. Int. J. Radiat. Oncol. Biol. Phys..

[11] Hardcastle I.R., Rowlands M.G., Moreno Barber A., Grimshaw R.M., Mohan M.K., Nutley B.P., Jarman M. (1999). Inhibition of protein prenylation by metabolites of limonene. Biochem. Pharmacol..

[12] Szczepanik A., Sobkowiak A. (2009). Utlenianie limonenu tlenem cząsteczkowym i nadtlenkiem wodoru. Wiadomości Chem..

[13] Gawarecka A., Wróblewska A., Pełech R. (2015). Limonene epoxidation on selected titanium-silicate catalyst. Tech. Issues.

[14] Duetz W.A., Bouwmeester H., Van Beilen J.B., Witholt B. (2003). Biotransformation of limonene by bacteria, fungi, yeasts, and plants. Appl. Microbiol. Biotechnol..

[15] Kołodziejczyk A. (2003). Naturalne Związki Organiczne.

[16] Sobkowiak A., Szczepanik A., Naróg D., Charczuk M. (2015). Oxidation of limonene with dioxygen catalyzed by 2,2’-bipyridyl manganese(II) and iron(II) complexes supported on a bentonite carrier. Przem. Chem..

[17] Stanciulescu M., Ikura M. (2007). Limonene ethers from tire pyrolysis oil. Part 2: Continuous flow experiments. J. Anal. Appl. Pyrolysis.

[18] Bicas J.L., Barros F.F.C., Wagner R., Godoy H.T., Pastore G.M. (2008). Optimization of R-(+)-α-terpineol production by the biotransformation of R-(+)-limonene. J. Ind. Microbiol. Biotechnol..

[19] Kraidman G., Mukherjee B., Hill J. (1969). Conversion of d-limonene into an optically active isomer of α-terpineol by a cladosporium species. Bacteriol. Proc..

[20] Cadwalleder K.R., Braddock R.J., Parish M.E., Higgins D.P. (1989). Bioconversion of (+)-limonene by pseudomonas gladioli. J. Food Sci..

[21] Tan Q., Day D., Cadwallader K. (1998). Bioconversion of R-(+)-limonene by *P. digitatum* (NRRL 1202). Process Biochem..

[22] Safety Data Sheet According to the Summary of EC 1907/2006. https://www.polychromal.com/images/downloads/en/safety-datasheets/duracolour_magenta-en.pdf.

[23] Zhang X., Wang T., Ma L., Chang J. (2008). Vacuum pyrolysis of waste tires with basic additives. Waste Manag..

[24] Danon B., Van Der Gryp P., Schwarz C.E., Görgens J.F. (2015). A review of dipentene (dl-limonene) production from waste tire pyrolysis. J. Anal. Appl. Pyrolysis.

[25] Rofiqul I.M., Haniu H., Rafiqul A.B.M. (2007). Limonene-rich liquids from pyrolysis of heavy automotive tire wastes. J. Environ. Eng..

[26] Piskorz J., Majerski P., Radlein D., Wik T., Scott D.S. (1999). Recovery of carbon black from scrap rubber. Energy Fuels.

[27] Ferrero G., Maniatis K., Buekens A., Bridgwater A.V. (1990). Pyrolysis and Gasification.

[28] Mazloom G., Farhadi F., Khorasheh F. (2009). Kinetic modeling of pyrolysis of scrap tires. J. Anal. Appl. Pyrolysis.

[29] Senneca O., Salatino P., Chirone R. (1999). Fast heating-rate thermogravimetric study of the pyrolysis of scrap tyres. Fuel.

[30] Cheung K.Y., Lee K.L., Lam K.L., Lee C.W., Hui C.W. (2011). Integrated kinetics and heat flow modelling to optimise waste tyre pyrolysis at different heating rates. Fuel Process. Technol..

[31] Suhanya M., Thirumarimurugan M., Kannadasan T. (2013). Recovery of oil from waste tyres using pyrolysis method: Review. IJRET.

[32] Dimitrov D.H., Dimitrov H. (2008). Method and Equipment for Whole Tyre Pyrolysis. U.S. Patent.

[33] Williams P.T., Bottrill R.P., Cunliffe A.M. (1998). Combustion of tyre pyrolysis oil. Process Saf. Environ. Prot..

[34] Pringle J.A. (2006). Microwave Pyrolysis Apparatus for Waste Tires. U.S. Patent.

[35] Undri A., Rosi L., Frediani M., Frediani P. (2011). Microwave pyrolysis of polymeric materials. Microwave Heating.

[36] Ludlow-Palafox C., Chase H.A. (2001). Microwave-induced pyrolysis of plastic wastes. Ind. Eng. Chem. Res..

[37] Landini L., de Araújo S.G., Lugão A.B., Wiebeck H. (2007). Preliminary analysis to BIIR recovery using the microwave process. Eur. Polym. J..

[38] Fix S.R. (1980). Microwave devulcanization of rubber. Elastomerics.

[39] Zanchet A., Carli L.N., Giovanela M., Crespo J.S., Scuracchio C.H., Nunes R.C.R. (2009). Characterization of microwave-devulcanized composites of ground SBR scraps. J. Elastomers Plast..

[40] Hong C.K., Isayev A.I. (2001). Continuous ultrasonic devulcanization of carbon black-filled NR vulcanizates. J. Appl. Polym. Sci..

[41] Tapale M., Isayev A.I. (1998). Continuous ultrasonic devulcanization of unfilled NR vulcanizates. J. Appl. Polym. Sci..

[42] Isayev A.I., Chen J., Tukachinsky A. (1995). Novel ultrasonic technology for devulcanization of waste rubbers. Rubber Chem. Technol..

[43] Yun J., Isayev A.I. (2003). Recycling of roofing membrane rubber by ultrasonic devulcanization. Polym. Eng. Sci..

[44] Levin V.Y., Kim S.H., Isayev A.I., Massey J., Meerwall E. (1995). Ultrasound devulcanization of sulfur vulcanized SBR crosslink density and molecular mobility. Ultrasound Devulcanization.

[45] Diao B., Isayev A.I., Levin V.Y. (1999). Basic study of continuous ultrasonic devulcanization of unfilled silicone rubber. Rubber Chem. Technol..

[46] Oh J.S., Isayev A.I. (2002). Ultrasonically treated polypropylene/ground tire rubber blends. Rubber Chem. Technol..

[47] Tang L., Huang H. (2004). An investigation of sulfur distribution during thermal plasma pyrolysis of used tires. J. Anal. Appl. Pyrolysis.

[48] Chen D.T., Perman C.A., Riechert M.E., Hoven J. (1995). Depolymerization of tire and natural rubber using supercritical fluids. J. Hazard. Mater..

[49] Park S., Gloyna E.F. (1997). Statistical study of the liquefaction of used rubber tyre in supercritical water. Fuel.

[50] Onsri K., Prasassarakich P., Ngamprasertsith S. (2010). Co-liquefaction of coal and used tire in supercritical water. Energy Power Eng..

[51] Ahmad N., Abnisa F., Daud W.M.A.W. (2016). Potential use of natural rubber to produce liquid fuels using hydrous pyrolysis-a review. RSC Adv..

[52] Kojima M., Tosaka M., Ikeda Y., Kohjiya S. (2005). Devulcanization of carbon black filled natural rubber using supercritical carbon dioxide. J. Appl. Polym. Sci..

[53] Jiang K., Shi J., Ge Y., Zou R., Yao P., Li X., Zhang L. (2013). Complete devulcanization of sulfur-cured butyl rubber by using supercritical carbon dioxide. J. Appl. Polym. Sci..

[54] Bouvier J.M., Charbel F., Gelus M. (1987). Gas-solid pyrolysis of tire wastes—Kinetics and material balances of batch pyrolysis of used tires. Resour. Conserv..

[55] Williams P.T., Taylor D.T. (1993). Aromatization of tyre pyrolysis oil to yield polycyclic aromatic hydrocarbons. Fuel.

[56] Pakdel H., Pantea D.M., Roy C. (2001). Production of dl-limonene by vacuum pyrolysis of used tires. J. Anal. Appl. Pyrolysis.

[57] Pakdel H., Roy C., Aubln H., Jean G., Coulombe S. (1991). Formation of dl-Limonene in used tire vacuum pyrolysis oils. Environ. Sci. Technol..

[58] Roy C., Darmstadt H., Benallal B., Amen-Chen C. (1997). Characterization of naphtha and carbon black obtained by vacuum pyrolysis of polyisoprene rubber. Fuel Process. Technol..

[59] Roy C., Chaala A., Darmstadt H. (1999). The vacuum pyrolysis of used tires end-uses for oil and carbon black products. J. Anal. Appl. Pyrolysis.

[60] Roy C., Labrecque B., de Caumia B. (1990). Recycling of scrap tires to oil and carbon black by vacuum pyrolysis. Resour. Conserv. Recycl..

[61] Bouvier J.M., Gelus M. (1986). Pyrolysis of rubber wastes in heavy oils and use of the products. Resour. Conserv..

[62] Williams P.T., Brindle A.J. (2003). Aromatic chemicals from the catalytic pyrolysis of scrap tyres. J. Anal. Appl. Pyrolysis.

[63] Williams P.T., Brindle A.J. (2002). Catalytic pyrolysis of tyres: Influence of catalyst temperature. Fuel.

[64] López A., de Marco I., Caballero B.M., Laresgoiti M.F., Adrados A., Aranzabal A. (2011). Catalytic pyrolysis of plastic wastes with two different types of catalysts: ZSM-5 zeolite and red mud. Appl. Catal. B Environ..

[65] Shah J., Rasul Jan M., Mabood F. (2008). Catalytic pyrolysis of waste tyre rubber into hydrocarbons via base catalysts. Iran. J. Chem. Chem. Eng..

[66] Roy C., Blanchette D., de Caumia B. Industrial scale demonstration of the pyrocyclingTM process for the conversion of biomass to biofuels and chemicals. Proceedings of the 1st World Conference on Biomass for Energy and Industry.

[67] Olazar M., Aguado R., Arabiourrutia M., Lopez G., Barona A., Bilbao J. (2008). Catalyst effect on the composition of tire pyrolysis products. Energy Fuels.

[68] Amutio M., Lopez G., Artetxe M., Erkiaga A., Alvarez J., Barbarias I., Olazar M. (2012). Valorisation of waste tires by pyrolysis over a FCC catalyst in a conical spouted bed reactor. Chem. Eng. Trans..

[69] Murena F., Garufi E., Gioia F. (1996). Hydrogenative pyrolysis of waste tyres: Kinetic analysis. J. Hazard. Mater..

[70] Benallal B., Roy C., Pakdel H., Chabot S., Poirier M.A. (1995). Characterization of pyrolytic light naphtha from vacuum pyrolysis of used tyres comparison with petroleum naphtha. Fuel.

[71] Oledzka E., Pyskło L., Sobczak M., Łuksa A. (2006). Piroliza zuzytych opon w aspekcie technicznym i ekonomicznym oraz uszlachetnianie otrzymywanych produktów. Polimery.

[72] Darmstadt H., Roy C., Kaliguine S. (1994). Inorganic components and sulphur compounds in carbon blacks from vacuum pyrolysis of used tires. Kautschuk und Gummi Kunststoffe.

[73] Darmstadt H., Roy C., Kaliaguine S. (1994). ESCA characterization of commercial carbon blacks and of carbon blacks from vacuum pyrolysis of used tires. Carbon.

[74] Yang J., Tanguy P.A., Roy C. (1995). Numerical model for the vacuum pyrolysis of scrap tires in batch reactors. AIChE J..

[75] Yang J., Tanguy P.A., Roy C. (1995). Heat transfer, mass transfer and kinetics study of the vacuum pyrolysis of a large used tire particle. Chem. Eng. Sci..

[76] Darmstadt H., Roy C., Kaliaguine S. (1995). Characterization of pyrolytic carbon blacks from commercial tire pyrolysis plants. Carbon.

[77] Leblanc J.L., Roy C., Mirmiran S., Benallal B., Schwerdtfeger A.E. (1996). The plasticizing properties of heavy oils obtained from the vacuum pyrolysis of used tires. Kautsch. Gummi Kunstst..

[78] Roy C., Rastegar A., Kaliaguine S., Darmstadt H., Tochev V. (1995). Physicochemical properties of carbon blacks from vacuum pyrolysis of used tires. Plast. Rubber Compos. Process. Appl..

[79] Mirmiran S., Pakdel H., Roy C. (1992). Characterization of used tire vacuum pyrolysis oil: Nitrogenous compounds from the naphtha fraction. J. Anal. Appl. Pyrolysis.

[80] Roy V., de Caumia B., Roy C. (1992). Development of a gas-cleaning system for a scrap-tire vacuum-pyrolysis plant. Gas Sep. Purif..

[81] Pakdel H., Roy C. (1994). Simultaneous gas chromatographic—Fourier transform infrared spectroscopic—Mass spectrometric analysis of synthetic fuel derived from used tire vacuum pyrolysis oil, naphtha fraction. J. Chromatogr. A.

[82] Mikulova Z., Honus S., Juchelkova D., Strakoś V. (2012). Laboratory and pilot research of pyrolysis process. Trans. VSB.

[83] Mastral A.M., Murillo R., Callén M.S., García T., Snape C.E. (2000). Influence of process variables on oils from tire pyrolysis and hydropyrolysis in a swept fixed bed reactor. Energy Fuels.

[84] Mastral A.M., Murillo R., Callen M.S., Garcia T. (2000). Optimisation of scrap automotive tyres recycling into valuable liquid fuels. Resour. Conserv. Recycl..

[85] Williams P.T., Brindle A.J. (2003). Temperature selective condensation of tyre pyrolysis oils to maximise the recovery of single ring aromatic compounds. Fuel.

[86] Wolfson D.E., Beckman J.A., Walters J.G., Bennett D.J. (1969). Destructive Distillation of Scrap Tires.

[87] López F.A., Centeno T.A., Alguacil F.J., Lobato B., Urien A. (2013). The grauthermic-tyres process for the recycling of granulated scrap tyres. J. Anal. Appl. Pyrolysis.

[88] González J.F., Encinar J.M., Canito J.L., Rodríguez J.J. (2001). Pyrolysis of automobile tyre waste. Influence of operating variables and kinetics study. J. Anal. Appl. Pyrolysis.

[89] Laresgoiti M.F., Caballero B.M., De Marco I., Torres A., Cabrero M.A., Chomón M.J. (2004). Characterization of the liquid products obtained in tyre pyrolysis. J. Anal. Appl. Pyrolysis.

[90] De Marco Rodriguez I., Laresgoiti M.F., Cabrero M.A., Torres A., Chomón M.J., Caballero B. (2001). Pyrolysis of scrap tyres. Fuel Process. Technol..

[91] Zabaniotou A.A., Stavropoulos G. (2003). Pyrolysis of used automobile tires and residual char utilization. J. Anal. Appl. Pyrolysis.

[92] Bouvier J.M., Farhadi F., Gelus M. (1983). A new method for upgrading rubber wastes. Int. Chem. Eng..

[93] Yanik J., Yüksel M., Salam M., Olukcu N., Bartle K., Frere B. (1995). Characterization of the oil fractions of shale oil obtained by pyrolysis and supercritical water extraction. Fuel.

[94] Hall W.J., Zakaria N., Williams P.T. (2009). Pyrolysis of latex gloves in the presence of Y-zeolite. Waste Manag..

[95] Williams P.T., Brindle A.J. (2003). Fluidised bed pyrolysis and catalytic pyrolysis of scrap tyres. Environ. Technol. UK.

[96] Karthikeyan S., Sathiskumar C., Moorthy S.R. (2012). Effect of process parameters on tire pyrolysis: A review. J. Sci. Ind. Res..

[97] Larsen J.W. (1976). Conversion of Waste Rubber to Fuel and Other Useful Products. U.S. Patent.

[98] Grzywa E., Molenda J. (2000). Technologia Podstawowych Syntez Organicznych.

[99] Lopez G., Artetxe M., Maider A., Haritz A., Olazar M., Elordi G. A conical spouted bed reactor for the valorisation of waste tires. Proceedings of the 13th International Conference on Fluidization New Paradigm in Fluidization Engineering.

[100] Arabiourrutia M., Lopez G., Elordi G., Olazar M., Aguado R., Bilbao J. (2007). Product distribution obtained in the pyrolysis of tyres in a conical spouted bed reactor. Chem. Eng. Sci..

[101] Bajus M., Olahová N. (2011). Thermal conversion of scrap tyres. Pet. Coal.

[102] Dai X., Yin X., Wu C., Zhang W., Chen Y. (2001). Pyrolysis of waste tires in a circulating fluidized-bed reactor. Energy.

[103] Conesa J.A., Martín-Gullón I., Font R., Jauhiainen J. (2004). Complete study of the pyrolysis and gasification of scrap tires in a pilot plant reactor. Environ. Sci. Technol..

[104] Islam M.R., Parveen M., Haniu H., Islam Sarker M.R. (2010). Innovation in pyrolysis technology for management of scrap tire a solution of energy and environment. Int. J. Environ. Sci. Dev..

[105] Islam M.R., Joardder M.U.H., Kader M.A., Islam Sarker M.R. Valorization of solid tire wastes available in Bangladesh by thermal treatment. Proceedings of the WasteSafe 2011—2nd International Conference on Solid Waste Management in the Developing Countries.

[106] Cunliffe A.M., Williams P.T. (1998). Composition of oils derived from the batch pyrolysis of tyres. J. Anal. Appl. Pyrolysis.

[107] Mkhize N.M., van der Gryp P., Danon B., Görgens J.F. (2016). Effect of temperature and heating rate on limonene production from waste tyre pyrolysis. J. Anal. Appl. Pyrolysis.

[108] Acevedo B., Barriocanal C. (2014). Fuel-oils from co-pyrolysis of scrap tyres with coal and a bituminous waste. Influence of oven configuration. Fuel.

[109] Alvarez J., Lopez G., Amutio M., Mkhize N.M., Danon B., van der Gryp P., Görgens J.F., Bilbao J., Olazar M. (2017). Evaluation of the properties of tyre pyrolysis oils obtained in a conical spouted bed reactor. Energy.

[110] Choi G.G., Jung S.H., Oh S.J., Kim J.S. (2014). Total utilization of waste tire rubber through pyrolysis to obtain oils and CO2 activation of pyrolysis char. Fuel Process. Technol..

[111] Choi G.G., Oh S.J., Kim J.S. (2017). Clean pyrolysis oil from a continuous two-stage pyrolysis of scrap tires using in-situ and ex-situ desulfurization. Energy.

[112] Choi G.G., Oh S.J., Kim J.S. (2016). Non-catalytic pyrolysis of scrap tires using a newly developed two-stage pyrolyzer for the production of a pyrolysis oil with a low sulfur content. Appl. Energy.

[113] Idris R., Chong C.T., Asik J.A., Ani F.N. (2020). Optimization studies of microwave-induced co-pyrolysis of empty fruit bunches/waste truck tire using response surface methodology. J. Clean. Prod..

[114] Kumar Singh R., Ruj B., Jana A., Mondal S., Jana B., Kumar Sadhukhan A., Gupta P. (2018). Pyrolysis of three different categories of automotive tyre wastes: Product yield analysis and characterization. J. Anal. Appl. Pyrolysis.

[115] Mkhize N.M., Danon B., Alvarez J., Lopez G., Amutio M., Bilbao J., Olazar M., van der Gryp P., Görgens J.F. (2019). Influence of reactor and condensation system design on tyre pyrolysis products yields. J. Anal. Appl. Pyrolysis.

[116] Song Z., Liu L., Yang Y., Sun J., Zhao X., Wang W., Mao Y., Yuan X., Wang Q. (2018). Characteristics of limonene formation during microwave pyrolysis of scrap tires and quantitative analysis. Energy.

[117] Song Z., Yang Y., Zhao X., Sun J., Wang W., Mao Y., Ma C. (2017). Microwave pyrolysis of tire powders: Evolution of yields and composition of products. J. Anal. Appl. Pyrolysis.

[118] Li S.Q., Yao Q., Chi Y., Yan J.H., Cen K.F. (2004). Pilot-scale pyrolysis of scrap tires in a continuous rotary kiln reactor. Ind. Eng. Chem. Res..

[119] Mirmiran S. (1994). Ph.D. Thesis.

[120] Chien J.C.W., Kiang J.K.Y. (1979). Polymer reactions-X thermal pyrolysis of poly(isoprene). Eur. Polym. J..

[121] Bhowmick A.K., Rampalli S., Gallagher K., Seeger R., McIntyre D. (1987). The degradation of guayule rubber and the effect of resin components on degradation at high temperature. J. Appl. Polym. Sci..

[122] Groves S.A., Lehrle R.S., Blazsó M., Székely T. (1991). Natural rubber pyrolysis: Study of temperature-and thickness-dependence indicates dimer formation mechanism. J. Anal. Appl. Pyrolysis.

[123] Ciochina O. (1997). Etude de L’influence de L’incorporation de L’huile Lourde Obtenue par Pyrolyse Sous Vide de Vieux Pneumatiques Sur Les Proprie´te´s des Bitumes Routiers.

[124] Roy C., Caumia B., de Pakdel H., Plante P., Blanchette D., Labrecque B. (1999). Vacuum pyrolysis of used tires, petroleum sludges and forestry wastes technological development and implementation perspectives. J. Anal. Appl. Pyrolysis.

[125] Aubin H.H. (1987). Memoire de Maitrise. Master’s Thesis.

[126] Gulzad A. (2011). Recycling and Pyrolysis of Scrap Tire.

[127] López G., Olazar M., Aguado R., Bilbao J. (2010). Continuous pyrolysis of waste tyres in a conical spouted bed reactor. Fuel.

[128] Kim S., Park J.K., Chun H.D. (1995). Pyrolysis kinetics of scrap tire rubbers. I: Using DTG and TGA. J. Environ. Eng. USA.

[129] Mkhize N.M., Danon B., van der Gryp P., Görgens J.F. (2019). Kinetic study of the effect of the heating rate on the waste tyre pyrolysis to maximise limonene production. Chem. Eng. Res. Des..

[130] Aguado R., Olazar M., Vélez D., Arabiourrutia M., Bilbao J. (2005). Kinetics of scrap tyre pyrolysis under fast heating conditions. J. Anal. Appl. Pyrolysis.

[131] Uzun B.B., Yaman E. (2014). Thermogravimetric characteristics and kinetics of scrap tyre and *Juglans regia* shell co-pyrolysis. Waste Manag. Res..

[132] Teng H., Serio M.A., Wójtowicz M.A., Bassilakis R., Solomon P.R. (1995). Reprocessing of used tires into activated carbon and other products. Ind. Eng. Chem. Res..

[133] Oyedun A.O., Lam K.-L., Gebreegziabher T., Lee H.K.M., Hui C.-W. Optimization of Multi-Stage Waste Tyre Pyrolysis Process. Proceedings of the 20th European Symposium on Computer Aided Process Engineering, ESCAPE20.

[134] Cheung K.Y., Lee K.L., Lam K.L., Chan T.Y., Lee C.W., Hui C.W. (2011). Operation strategy for multi-stage pyrolysis. J. Anal. Appl. Pyrolysis.

[135] Al-Salem S.M., Lettieri P. (2010). Kinetic study of high density polyethylene (HDPE) pyrolysis. Chem. Eng. Res. Des..

[136] Lopez G., Aguado R., Olazar M., Arabiourrutia M., Bilbao J. (2009). Kinetics of scrap tyre pyrolysis under vacuum conditions. Waste Manag..

[137] Chen J.H., Chen K.S., Tong L.Y. (2001). On the pyrolysis kinetics of scrap automotive tires. J. Hazard. Mater..

[138] Islam M.R., Haniu H., Fardoushi J. (2009). Pyrolysis kinetics behavior of solid tire wastes available in Bangladesh. Waste Manag..

[139] Quek A., Balasubramanian R. (2009). An algorithm for the kinetics of tire pyrolysis under different heating rates. J. Hazard. Mater..

[140] Olazar M., Lopez G., Arabiourrutia M., Elordi G., Aguado R., Bilbao J. (2008). Kinetic modelling of tyre pyrolysis in a conical spouted bed reactor. J. Anal. Appl. Pyrolysis.

[141] Leung D.Y.C., Wang C.L. (1998). Kinetic study of scrap tyre pyrolysis and combustion. J. Anal. Appl. Pyrolysis.

[142] Ucar S., Karagoz S., Ozkan A.R., Yanik J. (2005). Evaluation of two different scrap tires as hydrocarbon source by pyrolysis. Fuel.

